# The Clinical Significance of Genetic Variation in Ovarian Cancer

**DOI:** 10.3390/ijms241310823

**Published:** 2023-06-28

**Authors:** Dongjo Ban, Stephen N. Housley, John F. McDonald

**Affiliations:** Integrated Cancer Research Center, School of Biological Sciences, Georgia Institute of Technology, 315 Ferst Drive, Atlanta, GA 30332, USA; dongjoban@gatech.edu (D.B.); nickhousley@gatech.edu (S.N.H.)

**Keywords:** ovarian cancer, cancer progression, genomic profiles, tumor mutational burden, chromosomal alterations

## Abstract

Genetic variation is a well-known contributor to the onset and progression of cancer. The goal of this study is to provide a comprehensive examination of the nucleotide and chromosomal variation associated with the onset and progression of serous ovarian cancer. Using a variety of computational and statistical methods, we examine the exome sequence profiles of genetic variants present in the primary tumors of 432 ovarian cancer patient samples to compute: (1) the tumor mutational burden for all genes and (2) the chromosomal copy number alterations associated with the onset/progression of ovarian cancer. Tumor mutational burden is reduced in the late vs. early stages, with the highest levels being associated with loss-of-function mutations in DNA-repair genes. Nucleotide variation and copy number alterations associated with known cancer driver genes are selectively favored over ovarian cancer development. The results indicate that genetic variation is a significant contributor to the onset and progression of ovarian cancer. The measurement of the relative levels of genetic variation associated with individual ovarian cancer patient tumors may be a clinically valuable predictor of potential tumor aggressiveness and resistance to chemotherapy. Tumors found to be associated with high levels of genetic variation may help in the clinical identification of high-risk ovarian cancer patients who could benefit from more frequent monitoring.

## 1. Introduction

The onset and progression of ovarian cancer, and indeed all cancers, is a complex, multi-faceted process; however, at its root, cancer is a genetic disease [1]. The ultimate cause of all cancers is the heritable changes in the sequence, structure, and/or packaging (epigenetics) of DNA. While inherited predispositions to the development of cancer are attributable to inherited genetic variants encoded in the germline (e.g., breast cancer genes (*BRCA*) [2]), most cancers are associated with spontaneous mutational events occurring in the somatic tissues [3]. Genomic-level analyses of the onset and progression of ovarian and other cancers over the last decade have contributed significantly to an ever-expanding understanding of the molecular processes underlying the disease [1,4]. This has led, in some instances, to the identification of previously unrecognized molecular sub-types of ovarian and other cancers [5,6,7] and the subsequent development of a variety of targeted gene therapies personalized for patients with tumors displaying the appropriate genetic signatures [8,9].

The importance of genetic variation to the onset and progression of ovarian and other cancers, however, extends well beyond its utility as a potential classifier of cancer sub-types or as a pool of potential candidates for targeted gene therapy. Geneticists long ago recognized that the emergence of new adaptive traits in natural populations is directly correlated with the level of functionally significant genetic variation present (i.e., “Fisher’s fundamental theorem of natural selection” [10]); this same principle applies to the ability of tumors to develop (evolve) into more aggressive phenotypes, including those gaining resistance to cancer therapies [11,12]. As is the case in natural populations, the levels of genetic variation associated with tumors can be influenced by both directed and random processes. For example, directional selection for more rapidly dividing sub-clones, or for sub-clones more resistant to specific chemotherapies, can result in significant changes in the levels and composition of genetic variation in tumors [13]. However, undirected processes, such as the random sloughing-off of cancer cells from primary tumors and their sub-sequent spread to other organs, may lead to significant changes in the genetic make-up of derived metastatic tumors [14]. Additional random sampling processes associated with tumor onset and early development may also have a significant impact on tumor progression [15,16].

We report here, the results of a detailed examination of genetic variation associated with the onset and progression of serous ovarian cancer (OC), the most prevalent and lethal sub-type [17]. We detected significant changes in the levels and patterns of both nucleotide and chromosomal variation throughout ovarian cancer onset and development. We present evidence that most of these changes are driven by selection and may constitute useful predictors of a patient’s prognosis and response to both chemo- and immuno-therapies.

## 2. Results

### 2.1. High TMB Levels Are Rare in the OC Cohort

**Figure 1:**
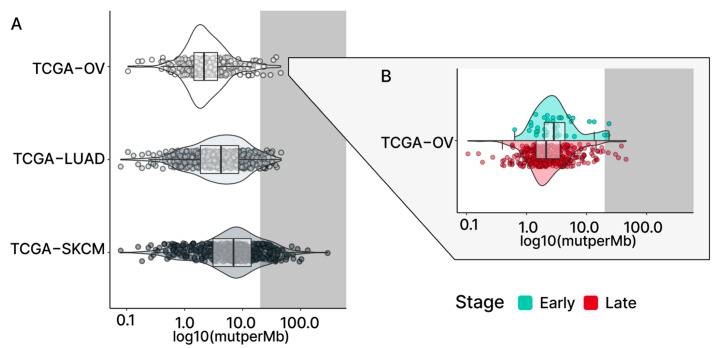
TMBs in TCGA cohorts. (**A**) The violin plots present the TMB levels of three selected TCGA study cohorts. TCGA-OV (serous ovarian cancer) shows a relatively lower median TMB compared to the TCGA-LUAD (lung adenocarcinoma) and TCGA-SKCM (skin cutaneous melanoma) cancer types. In TCGA-OV, only a few samples with high-tier TMB (grey; >20 mutations/Mb) can be found compared to the other two cohorts. The stage-level TMB for TCGA-OV is shown in (**B**), with the early stage (light blue) showing a slightly higher median TMB compared to the late stage (red).

To study the level of genetic variations in serous ovarian cancer (OC), 432 somatic mutation profiles from The Cancer Genome Atlas (TCGA) OC cohort were downloaded from Genomic Data Commons (GDC) [18]. A commonly used metric of variation is tumor mutation burden (TMB), which is defined as the number of non-synonymous substitutions and short indels per megabase of DNA (mutations/Mb) [19]. While the median TMB for OC relative to most other cancer types is low, a minority of our OC patient samples falls in the high TMB range [20,21,22] (Figure 1A).

### 2.2. Early-Stage OC Patients Are Associated with Slightly Higher Levels of TMB

To assess potential differences in the levels of TMB between the stages of OC, the 432 samples were sub-grouped as early-stage (stages I, II) or late-stage (stages III, IV) based on the FIGO staging system [23]. This resulted in the classification of 38 of our samples as early-stage and 394 samples as late-stage. The relative paucity of early-stage samples reflects the fact that the disease is typically asymptomatic at early stages of tumorigenesis and, thus, difficult to detect [24].

**Figure 2:**
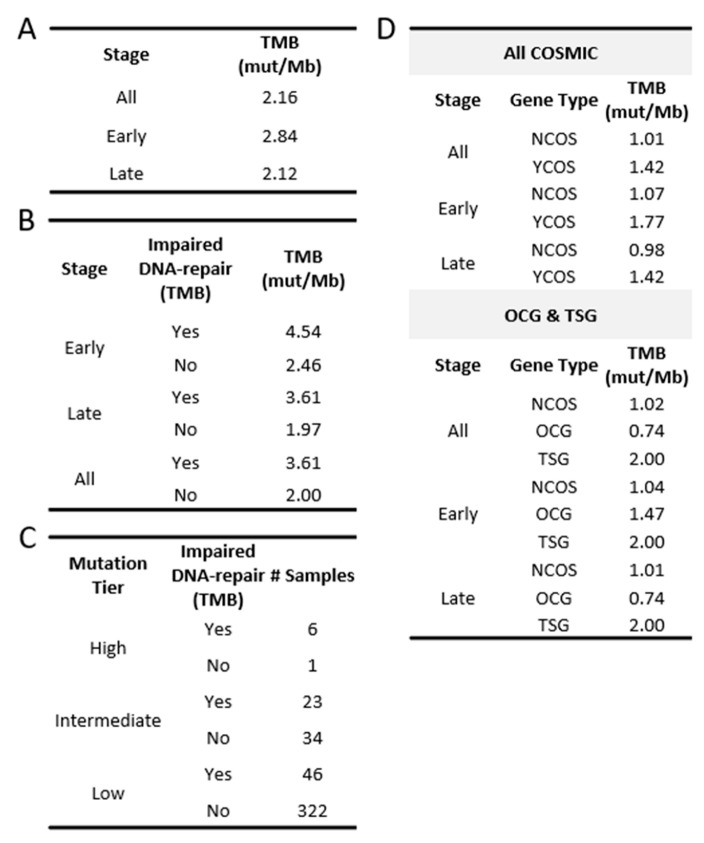
TMB in TCGA-OV. (**A**) TMB found in the OC cohort and in each stage group. (**B**) TMB found in those with impaired DNA-repair genes and those without. (**C**) Number of OC patients belonging to each mutation tier and their DNA-repair gene status. (**D**) TMB found in COSMIC genes. (YCOS = COSMIC; NCOS = non-COSMIC).

In comparison to the median TMB found in terms of the overall TCGA-OC cohort and the late-stage patients, the early-stage patient group was found to have a slightly higher median TMB (all: 2.16 mutations/Mb; early: 2.84 mutations/Mb; late: 2.12 mutations/Mb; Figure 1B and Figure 2A). However, the significance of these observed differences in the medians is uncertain due to the small size of our early-stage samples. To address this question, we adopted the previously described Bayesian analysis approach [25,26,27] to assess the fixed-effect of the stage on TMB. This approach considers the uncertainty in settings with smaller sample sizes and enables us to make more subtle inferences. The results of the Bayesian analysis demonstrated that individuals in the late-stage group, relative to the early-stage group, had a reduced level of TMB (estimate: −1.48; 95% CI: [−3.16–−0.31]; Table S1), which strengthened our confidence in the previously observed difference in medians.

### 2.3. Lack of Correlation between TMB and Age or Grade of OC Patients

**Figure 3:**
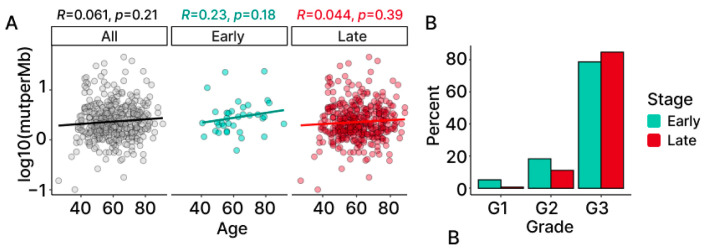
TMB in association with age and grade. (**A**) Correlation between TMB and age computed for all (black), early-stage (light blue) and late-stage (red), with the coefficients and the *p*-values. (**B**) Distribution of grade information for TCGA-OV using a percentage of early- and late-stage samples belonging to each grade (G1: low; G2: intermediate; G3: high).

To explore whether patient age may be influencing TMB, we computed a Spearman correlation between the two variables. The results demonstrate no significant association between patient age and TMB overall, nor within the individual stage groups (Figure 3A).

A similar approach was taken to examine the possibility that differences in TMB may be associated with tumor grade. While this analysis was limited by the lack of diversity found in the grade of the patients (Figure 3B), fixed-effects modeling provides evidence that grade does not affect the levels of TMB in our OC patient samples (Table S1).

### 2.4. Higher Levels of TMB Are Associated with LOF Mutations in DNA-Repair Genes

**Figure 4:**
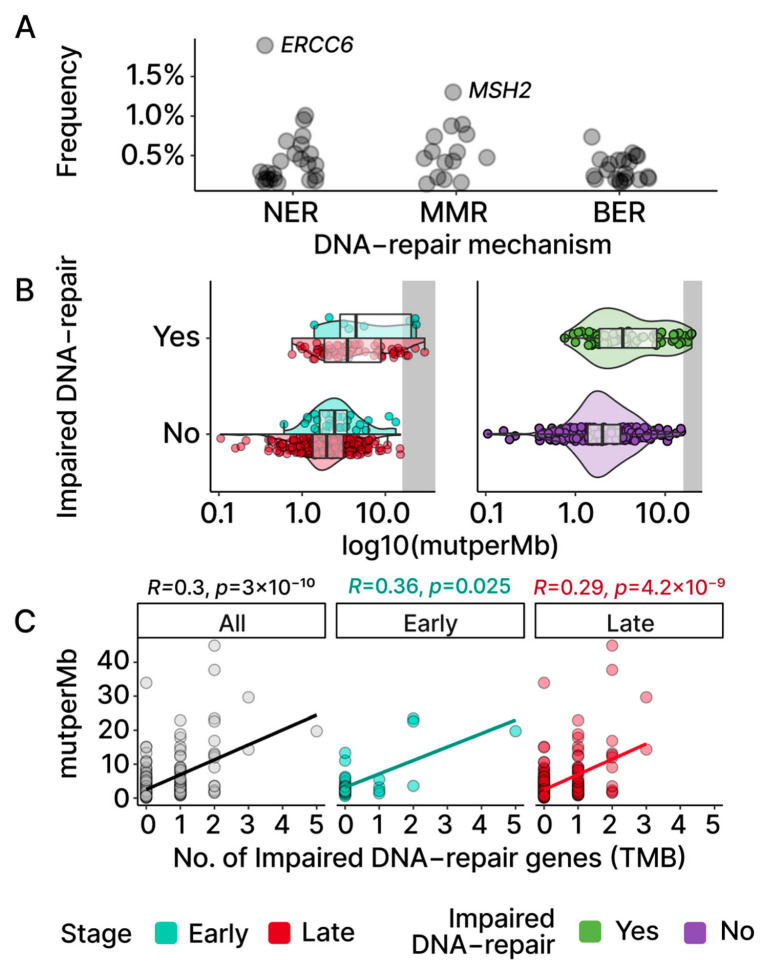
TMBs and DNA-repair genes. (**A**) Each point represents a gene involved in NER, MMR, and BER, with the frequencies of its mutations observed in TCGA-OV. Aside from *ERCC6* and *MSH2*, other DNA-repair genes display low frequencies. (**B**) Samples are grouped based on whether an impaired DNA-repair gene was found based on REVEL scores (Section 4). The violin and box plots visualize both TMB distributions and group medians. (**C**) Spearman correlation between the numbers of impaired DNA-repair genes found in samples and TMBs. Both stages display significant and moderate positive correlations that can also be observed at the cohort level.

Previous studies have reported an association between the presence of mutations in DNA-repair genes and high levels of TMB in a multitude of cancers [22,28,29,30]. To explore if such an association is apparent in our early- and late-stage OC patient samples, we selected a group of 70 genes (Table S2) that were previously identified as being involved in nucleotide excision repair (NER), base excision repair (BER), and mismatch repair (MMR) [28]. Utilizing an established in silico method, REVEL, for the identification of pathogenic loss-of-function (LOF) mutations [31], we searched for potentially damaging mutations in DNA-repair genes in each of our patient samples. As shown in Figure 4A, the frequency of LOF mutations for each individual DNA-repair gene was low across the OC cohort.

We next partitioned the patient samples into those with a LOF mutation in at least one of the aforementioned DNA-repair genes and those without. This resulted in a total of 75 samples (early: 8; late: 67) with at least one DNA-repair gene associated with LOF mutation versus 357 samples without any mutations in the set of DNA-repair genes (early: 30; late: 327). The group TMB medians based on the DNA-repair status exhibited a higher TMB in those with at least one impaired DNA-repair gene (Figure 2B and Figure 4B; early: 4.54 vs. 2.46; late: 3.61 vs. 1.97). Additionally, the effect of the number of impaired DNA-repair genes on the TMB level was assessed using the Spearman correlation. A moderate positive correlation (early: *p* = 0.025; late: *p* = 4.2 × 10^−9^; Figure 4C) was observed in both the early- and late-stage groups, indicating a positive association between the level of dysfunctional DNA-repair mechanisms and the TMB levels in OC patients.

Although the median TMB for OC is considered low relative to other cancer types, some OC patients exhibited high TMB levels (Figure 1A; grey-shaded). To further explore the possibility that mutations in DNA-repair genes may be affecting the levels of TMB in OC tumors, patient samples were assigned to TMB tiers based on previously defined TMB criteria [20,21] (low: n < 5; intermediate: 5 < n < 20; high: n > 20). Seven OC samples were grouped into the high tier, fifty-seven samples into the intermediate tier, and three hundred and sixty-eight into the low TMB tier (Figure 2C). Despite the low number of samples in the high tier, six out of seven samples (86%) were found to have LOF mutations in at least one DNA-repair gene. In comparison, the intermediate and the low TMB tier samples displayed relatively fewer LOF mutations in DNA-repair genes (intermediate 40%; low 12.5%). Collectively, our results indicate that LOF mutations in DNA-repair genes contribute to TMB in both early- and late-stage OC.

### 2.5. Cancer Driver Genes Display Higher Levels of TMB in OC

Since its inception, COSMIC has provided valuable insights into genes believed to be involved in cancer onset and progression [32]. The Cancer Gene Consensus (CGC) component of COSMIC identified 723 cancer-associated genes through its curation process. Oncogenes (OCGs) and tumor-suppressor genes (TSGs) each consists of 243 genes when fusion genes and those classified as belonging to both sets (OCGs and TSGs) are omitted.

When all COSMIC genes (723), including those identified to be both OCG and TSG, were considered collectively, the TMBs found in the COSMIC genes displayed a higher median TMB in comparison to those in the non-COSMIC genes (YCOS: 1.42; NCOS: 1.01). This difference was maintained in both early- and late-stage samples when considered separately (Figure 2D). Moreover, the median TMB for TSGs was higher than the TMB for the OCGs sub-group (OCG: 0.74; TSG: 2.0). Bayesian analysis (MLE, 95% credible interval) corroborated these findings and suggested that COSMIC genes are positively associated with TMB levels (YCOS: 0.51, CI: 0.33–0.69; OCG: 0.44, CI: 0.20–0.68; TSG: 0.75, CI: 0.51–0.99; Table S1).

### 2.6. Mutations in Cancer Driver Genes Are Selectively Favored during Cancer Onset and Progression

Having established a difference in the TMB level between COSMIC and non-COSMIC genes, we next explored the extent to which these differences may reflect the selection for mutations favorable for tumor growth. One computational approach long used to detect evidence of protein-level selection in the field of population genetics is the ratio between the non-synonymous substitutions (*dN*) and synonymous substitutions (*dS*) [33]. A modification of this approach has recently been adapted for use in the analysis of genetic changes associated with cancer [34]. Using this approach, we computed *dN*/*dS* ratios in our OC patient samples to look for evidence of selection on the protein level. A *dN*/*dS* ratio greater than 1.00 indicates positive selection, where nucleotide changes favorable to cancer growth are observed at higher frequencies than the changes at neutral sites. Likewise, a ratio of less than 1.00 indicates negative selection, where nucleotide changes unfavorable to cancer growth are selected against and, thus, observed at lower frequencies than the changes at neutral sites. Ratios at or near 1.00 are indicative of nucleotide changes having little-to-no effect on cancer development.

Table 1 presents *dN*/*dS* ratio estimates for COSMIC and non-COSMIC genes. Overall, COSMIC genes display significantly higher *dN*/*dS* estimates than non-COSMIC genes (YCOS: 1.20, CI: 1.11–1.31; NCOS: 1.05, CI: 1.03–1.08). The stage-specific ratio estimates display a similar trend (early-YCOS: 1.23, CI: 0.97–1.58; early-NCOS: 1.01, CI: 0.95–1.08; late-YCOS: 1.19, CI: 1.09–1.30; late-NCOS: 1.05, CI: 1.03–1.08). These findings are indicative of positive selection for COSMIC gene mutations, with no evidence of selection for mutations in non-COSMIC genes during tumorigenesis.

OCGs were found to display slightly higher *dN*/*dS* ratio estimates (*dN*/*dS* = 1.61) than TSGs (=1.24) in the early stages of cancer development. However, these differences are likely insignificant given the overlap in the 95% confidence intervals. This trend was dramatically reversed in late-stage tumors where there is strong evidence that TSG LOF mutations were more positively selected (*dN*/*dS* = 1.49) than those associated with OCG (*dN*/*dS* = 1.01), largely due to a significant reduction in the *dN*/*dS* ratio estimates for OCGs in late-stage cancers. When nucleotide substitutions are broken down into sub-types, it becomes clear that the reduction in the OCG *dN*/*dS* ratio estimates in late-stage tumors is largely attributable to a strong negative selection against mutations mapping to splice sites (OCG: 0.38, CI: 0.19–0.74). Evidence of negative selection against splice site mutations was also evident for non-COSMIC genes in both stage groups of OC development.

### 2.7. LOF Mutations in TP53 Are Detected in a Majority of Both Early- and Late-Stage OC Patients

**Figure 5:**
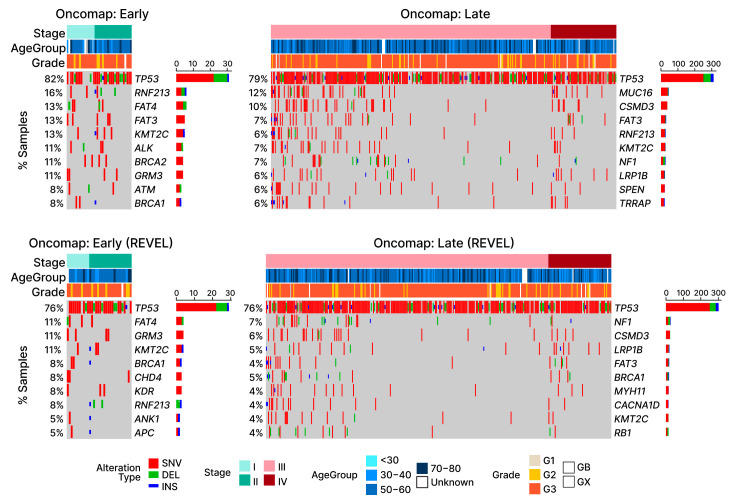
Oncomap of LOF mutations in TCGA-OV. Four oncomaps visualize the mutations observed in OC patients, with the bottom row of graphs displaying only potentially damaging mutations according to the REVEL scores. Depending on the type of alteration, samples are either marked with red (SNV: single nucleotide variant), green (DEL: short deletion), or blue (INS: short insertion). For visualization purposes, samples without any substitutions in the listed genes are omitted. Aside from *TP53*, other cancer driver genes (COSMIC) display relatively lower frequencies, as shown by the stacked bar plots next to the gene names.

In light of the evidence of positive selection for COSMIC gene mutations in our OC patient samples, we were interested in identifying the cancer driver genes most frequently involved. Figure 5 displays the ten most common COSMIC gene mutations present in our early- and late-stage OC samples. Consistent with previous findings [35], we found that mutations in the *TP53* gene are by far the most common genetic mutation shared across all OC patient samples. Upon screening for potentially deleterious mutations, more than 70% of mutations in both early- and late-stage ovarian cancer groups were associated with either single nucleotide variants or short indels, known to disrupt the function of *TP53* [36]. The frequencies of the mutations associated with the other nine most frequently mutated COSMIC genes were much lower those of *TP53*, ranging from 4% to 11% depending on the stage group.

### 2.8. OC Patient Tumors Display a Large Number of Chromosomal Alterations

In addition to SNVs and short indels, cancers have been associated with amplifications or deletions of genetic material, collectively referred to as copy number alterations (CNAs) [37]. To determine the extent to which CNAs associate with early- and late-stage OC patients, 570 gene-level copy number (CN) profiles of OC patients were retrieved from the GDC. Gene-level CNA estimates for the individual OC samples were obtained by analyzing the changes in the copy numbers of chromosomal segments through GISTIC2.0. The gains (AMP) and losses (DEL) were assigned positive and negative values of 1.00, respectively, regarding genes belonging to the affected chromosomal segment.

**Figure 6:**
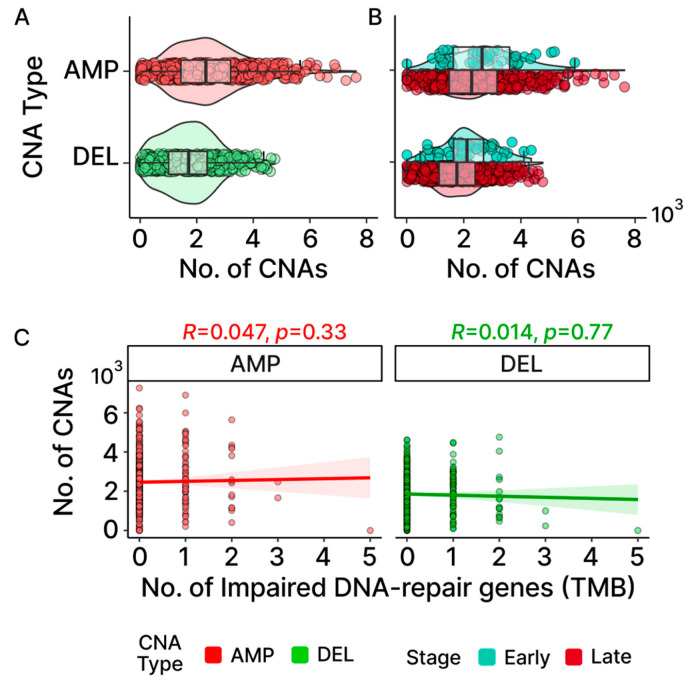
Patterns of CNAs in TCGA-OV. Distribution of gene-level gains (AMP) and losses (DEL) found in samples are shown in (**A**) CNAs at the cohort level and (**B**) early- and late-stage groups. For AMP, there are outlier samples that clearly distinguish themselves further away from the box plot whiskers compared to DEL. (**C**) Neither AMP nor DEL displays any association between the number of impaired DNA-repair genes linked to TMB and the number of CNAs found in OC according to the Spearman correlation.

The medians observed for both gain and loss events in OC reveal that CNAs affect approximately 20% of the protein-coding genes, with AMP displaying more observed events than DEL (Figure 6A; 2323 AMP + 1784 DEL = 3962 CNAs/20,352 total protein-coding genes = 20.1%). Bayesian analysis provided evidential support that the early-stage OC group exhibited higher levels of CNAs consistent with the pattern previously exhibited in the TMB levels (Figure 6B; Table S3). Additionally, outlier patients displaying elevated levels of CNAs were also detected, with CNAs exhibiting a greater level of distinction in AMP. The CNA outliers did not overlap with the TMB outliers and were not found to be associated with more frequent mutations in DNA-repair genes associated with TMB, indicating that independent molecular processes are likely involved (Figure 6C).

### 2.9. CNAs Are Not Associated with Potentially Damaging Mutations Found in DNA-Repair and Cell-Division Genes

**Figure 7:**
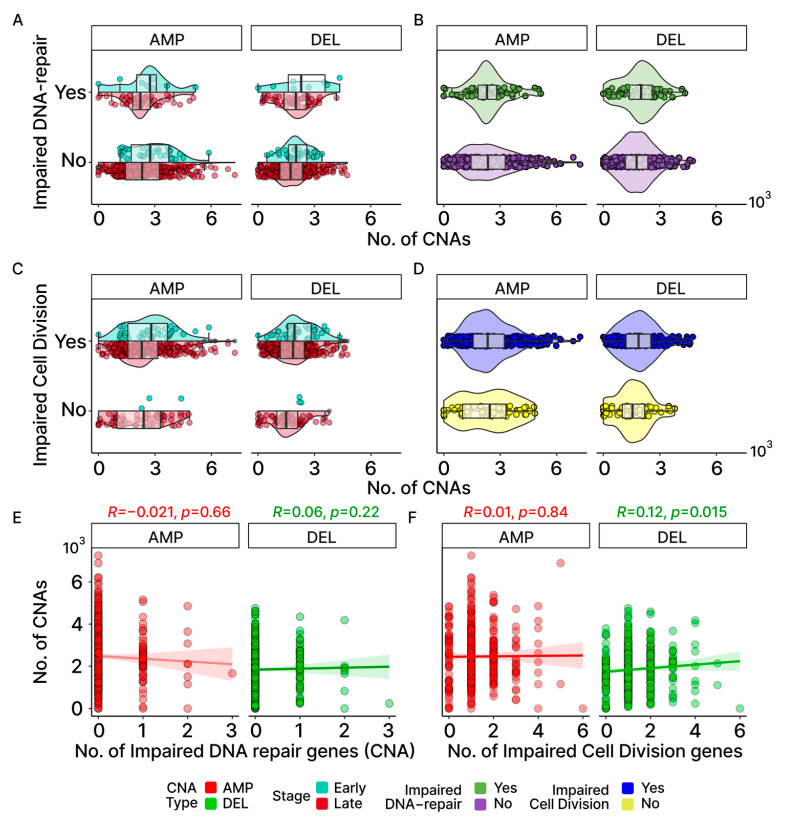
Association between CNAs and DNA-repair/cell-division genes. (**A**,**B**) Comparison of gene-level gains (AMP) and losses (DEL) between those with and without impaired DNA-repair genes involved in HR and NHEJ by stage (**A**) and overall (**B**). (**C**,**D**) Same comparison as (**A**,**B**) but for those with and without impaired cell-division (CD) genes. As most early-stage samples harbored at least one potentially pathogenic CD gene mutation, no violin or bar plot is displayed for the early-stage group in (**C**). (**E**,**F**) Spearman correlations between the number of impaired genes and the number of CNAs show no notable trend for DNA-repair and cell-division genes.

**Figure 8:**
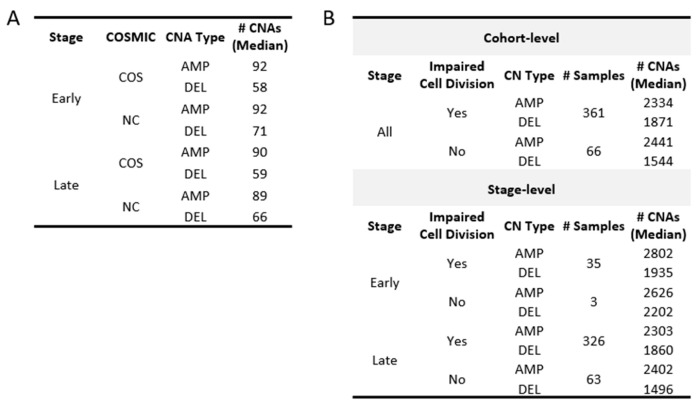
Median CNAs in groups based on the COSMIC genes and status of the cell-division genes. (**A**) Summarizes the number of gains (AMP) and losses (DEL) found in COSMIC genes. CNAs in non-COSMIC genes are also shown for comparison. (**B**) Samples are grouped based on whether they were found with at least one impaired cell-division (CELLDIV) gene. The number of samples, as well as the CNA group median, is displayed.

In an effort to identify molecular mechanisms potentially underlying the CNAs associated with our OC patients, we first looked for an association between these chromosomal events and mutations in the genes previously associated with CNA events. Earlier studies have demonstrated that CNAs may arise from errors during the process of homologous recombination (HR) or non-homologous end joining (NHEJ) [38]. Additionally, mutations in genes that are linked to cell division have previously been associated with CNA events [39,40]. To explore the possibility that mutations in these genes may be generally contributing to the elevated occurrences of CNAs observed in OC, we first independently filtered the list of all known DNA-repair genes for those associated with recombination [28,32] (Table S4). Figure 7A,B show little difference between the number of CNAs between patients with and without LOF mutations in the genes involved with HR and NHEJ. The group medians and fixed-effects with their credible intervals (Tables S3 and S5) suggest that there is no significant difference. For cell-division genes, only three samples in the early stage were found without any impaired cell-division genes. As such, the stage was not considered when assessing group median and fixed-effects, despite the early stage showing a higher number of CNAs than late stage for AMP in the OC group with impaired cell-division genes (Figure 7C,D). While the fixed-effects suggest that both CNA-type and impaired cell-division genes are significant in affecting the number of CNAs according to the credible intervals (CNA-Type (DEL): −629.5, 95% CI: −784.1–−475.3; impaired cell division (Yes): 252.5, 95% CI: 45.9–463; Table S3), the group medians indicate that a CNA difference due to impaired cell-division genes applies only to DEL (Figure 8A,B). Additionally, Spearman correlations for both gene groups further insinuate their roles may not be significant at the cohort level for OC based on their coefficients and their associated p-values (Figure 7E,F). It is important to note that although our results suggest that LOF mutations in these DNA-repair and cell cycle control genes are not generally associated with the frequency of CNA events across all patients, this does not eliminate the possibility that mutations in any one or more of these genes may have significant effects on individual patients.

### 2.10. Cancer-Associated Genes in Potential Chromosomal Driver Regions

**Figure 9:**
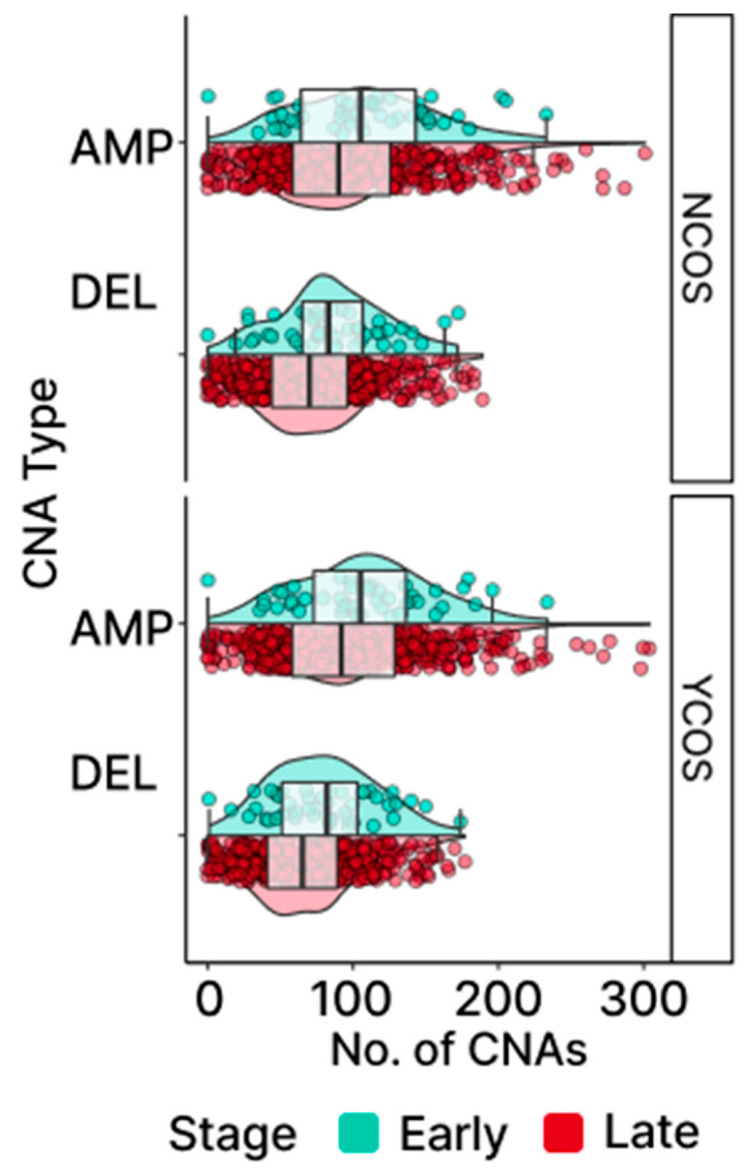
Distribution of CNAs in COSMIC genes. Gains (AMP) and losses (DEL) are shown for COSMIC (COS) and non-COSMIC (NC) genes. Samples beyond the box plot whiskers are CNA outliers.

Our prior analysis of TMB levels showed that COSMIC genes harbor higher levels of TMB. Using a similar approach, CNA levels in cancer-associated genes, including OCG and TSG sub-sets, were assessed against non-COSMIC genes to determine if CNAs were preferentially associated with chromosomal regions containing COSMIC genes. The group medians (Figure 8A and Figure 9) and Bayesian fixed-effect estimation (Table S3) demonstrate that COSMIC genes do not carry more CNAs than non-COSMIC genes. This was also true when considering OCG and TSG sub-sets individually (Figure S1, Tables S3 and S6).

We next examined the CNA-affected regions identified as putative drivers of OC. Chromosomal regions can be identified as potential “drivers” of cancer by GISTIC2.0, which incorporates both the amplitude (i.e., degree of amplification or deletion mapping to a chromosomal region) and the frequency of these events across patient samples. Utilizing this approach, we identified putative driver regions in our OC patients and looked for associations with COSMIC genes mapping to these driver regions.

**Figure 10:**
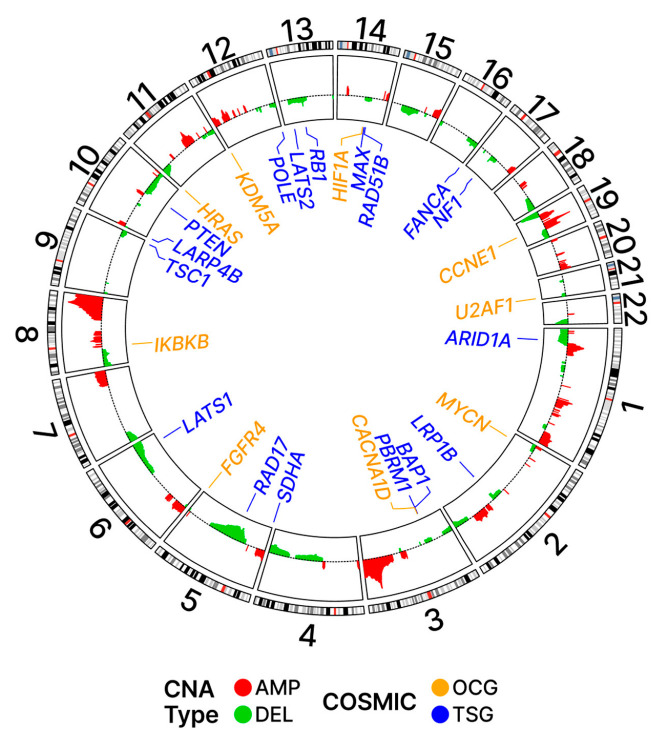
Ideogram with driver regions. Potential driver regions based on the G-scores and their adjusted *p*-values are shown on the inner panel with colors red and green for AMP and DEL, respectively. The loci for COSMIC genes are shown in the outer panel.

As shown in Figure 10, the chromosomal locations of the OC driver regions are widely distributed across the genome. Of the twenty-two pairs of autosomal chromosomes making up the human genome, all but seven (9, 16–18, 20–22) were found to be associated with putative driver regions containing COSMIC genes. Of the fifteen chromosomes associated with such driver regions, seven chromosomes were associated with both AMP and DEL events (1, 2, 5–8, 19); meanwhile, eight (AMP: 3, 12; DEL: 4, 10, 11, 13–15) were associated with either AMP or DEL events but not both (Table S7). Additionally shown in Figure 10, the chromosomal locations of the COSMIC genes are equally widely distributed across the genome. We were interested in looking at patterns of associations between the location of the COSMIC genes and regions identified as putative drivers of cancer.

Table 2 summarizes the proportions of the COSMIC genes relative to all genes associated with driver and non-driver regions. The results demonstrate that the proportion of COSMIC genes associated with amplification (AMP) events in driver regions is higher (4.32%) than the proportion in non-driver regions (3.24%). This finding is consistent with the hypothesis that AMP events are selectively favored in COSMIC genes in the context of OC. In contrast, the proportion of COSMIC genes associated with DEL events in driver regions is lower (2.68%) than the proportion in non-driver regions (3.34%), which aligns with the hypothesis that DEL events are selected against in COSMIC genes in OC.

These trends were more pronounced when only OCGs were considered. The proportion of OCGs associated with AMP events in driver regions was 1.77% while the proportion in non-driver regions was 1.10%; again, this is indicative of positive selection for AMP events associated with OCG sequences in driver regions. The proportion of OCGs associated with DEL events in driver regions was 1.03% while the proportion in non-driver regions was 1.16%; again, this is indicative of negative selection for DEL events associated with OCG sequences in driver regions.

When TSGs were considered separately, a reversal in the above trends was observed for AMP events. The proportion of TSGs associated with AMP events in driver regions was 0.71% while the proportion in non-driver regions was 1.21%; this is indicative of a negative selection for AMP events associated with TSG sequences in driver regions. The proportion of TSGs associated with DEL events in driver regions was 0.91% while the proportion in non-driver regions was 1.09%; this is indicative of a negative selection for DEL events associated with TSG sequences in driver regions.

The heights of the driver regions, also referred to as peaks, as shown in Figure 10, reflect not only the magnitude but also the frequency of the alterations in the driver regions.

Table 3 lists the OCGs and TSGs mapping to these peak regions. The TSGs were found to be associated with these regions more frequently than OCGs and most of these TSG-associated events were deletions (DEL). In contrast, the OCGs mapping to these frequently altered regions were most often associated with amplifications (AMP).

## 3. Discussion

In this study, we examined changes in the levels and patterns of genetic variation associated with the onset and progression of ovarian cancer utilizing data downloaded from the Genomic Data Commons (GDC). Two categories of genetic variants were examined: (1) single nucleotide substitutions/short nucleotide insertions/deletions (i.e., single nucleotide variants or SNVs) and (2) large regional amplifications/deletions in chromosomes, collectively referred to as copy number alterations (CNAs). To quantify the levels of SNVs in our patient samples, we employed the commonly used metric, tumor mutation burden (TMB), which is defined as the number of non-synonymous substitutions and short indels per megabase of DNA (mutations/Mb) [19]. For quantification of the variation of CNAs, we utilized the GISTIC2.0 algorithm [41], which assigns positive and negative values of 1 to gains (+1) and losses (−1) to gene sequences amplified or deleted in an affected chromosomal segment. Consistent with previous reports [22], we found that the median level of TMB in OC is low relative to most other cancer types. However, we also found that a significant minority of our OC patient tumors displayed what is generally considered high levels of TMB. Due to the relative paucity of CNA studies in other cancers, we were unable to perform comparative evaluations of CNAs. However, as was the case for TMB, outlier patients with tumors displaying relatively high levels of CNAs were also identified. The fact that the TMB outlier patients were distinct from those classified as CNA outliers suggests that the molecular mechanisms underlying these phenomena are distinct.

The existence of outlier OC patients displaying high levels of genetic variation (both in terms of TMB and CNAs) may have relevance to the challenge of identifying candidates for immunotherapy. While the clinical benefits of immunotherapy for some cancer patients have been dramatic [42], the overall response (20–40%) across all treated cancer patients has, thus far, been disappointing. This is especially true for OC patients, where positive response rates range from only 5% to 15% [43]. Consequently, substantial effort is currently being focused on identifying biomarkers that may accurately identify the cancer patients most likely to respond to immunotherapy. Among the more promising biomarkers identified, thus far, is TMB [44]. The underlying rationale is that patient tumors associated with exceptionally high levels of novel protein antigens are more likely to elicit an immune response. While the results of studies correlating levels of TMB and CNA with the response of OC patients to immunotherapies are not currently available, the fact that the frequency of outliers approximates the reported frequency of OC patients responding to immunotherapy is intriguing and warrants further study.

We found that levels of both SNVs and CNAs were significantly elevated in cancer samples relative to matched sets of normal tissues. Interestingly, the levels of both categories of these variants were also found to be higher in early-stage samples (FIGO stages I and II) relative to late-stage samples (FIGO stages III and IV). It is generally assumed that levels of genetic variation increase in cell lineages over time (i.e., the greater the number of cell divisions, the greater the accumulation of mutations) [45]. In so far as this assumption is valid, our results appear to be inconsistent with a commonly held view that early-stage cancers temporally precede late-stage cancers (i.e., the view that tumors temporally progress from early to late stages of development). This apparent inconsistency may be explained, at least in part, by the previously proposed hypothesis that there exist two clinically distinguishable classes of ovarian tumors [46]. According to this hypothesis, type-I tumors include low-grade serous cancers that rarely progress beyond the early stages of tumor development and, thus, may persist for extended periods of time. In contrast, type-II tumors include high-grade serous cancers and are believed to progress rapidly to advanced stages of tumor development (stages III and IV). Thus, if a significant number of our early-stage OCs are temporally older and less aggressive cancers, it may help explain our observation that levels of genetic variation are marginally higher in OC patients with an early-stage disease.

Errors associated with the underlying mechanisms of DNA replication are believed to be generally low [47]. However, if LOF mutations occur in genes encoding mechanisms of DNA-repair, it can result in a significant elevation in the levels of genetic variation [48]. Consistent with previous studies conducted in a variety of cancer types [22,28,29,30], we found that relative levels of TMB (i.e., nucleotide variation) were higher in the OC patients carrying LOF mutations in at least one DNA-repair gene, including the “outlier patients” displaying high levels of TMB. Thus, errors in the DNA-repair processes appear to be a significant factor in determining the levels of TMB in OC. Interestingly, no single DNA-repair gene was found to be predominantly responsible for this trend; this is consistent with the view that there are likely multiple alternative molecular pathways leading to the development of even the same cancer type [49].

We were unable to identify a significant correlation between the frequency of CNAs across patients and the mutations in genes previously implicated in changes in chromosome structure/number (i.e., genes regulating cell division, homologous recombination, non-homologous end joining). This suggests that many CNA events in OC are occurring randomly or are under the control of processes (or combinations of processes) yet to be fully identified.

Regardless of the molecular mechanisms underlying the generation of the new genetic variants associated with OC, the question remains as to whether this variation is of clinical significance, i.e., does this genetic variation provide a resource for the selection/evolution of more prolific cancer cells? We addressed this question independently for TMB levels and CNAs. To look for evidence of the selection for newly acquired nucleotide mutations in cancer driver genes, we utilized a method long-employed in the field of population genetics [50] that has been modified to identify nucleotide-level genetic variants selectively favored during cancer onset and development [34]. Using this approach, we computed *dN*/*dS* ratios (frequency of non-synonymous compared to the frequency of synonymous nucleotide mutations) regarding our OC patient samples to look for evidence of selection on the protein level. A ratio greater than 1.00 is indicative of positive selection, where nucleotide changes favorable to cancer growth are observed at higher frequencies than the changes at neutral sites. Likewise, a ratio less than 1.00 indicates negative selection, where nucleotide changes unfavorable to cancer growth are selected against and, thus, observed at lower frequencies than changes at neutral sites. Ratios (*dN*/*dS*) at or near 1.00 are indicative of nucleotide changes having little-to-no effect on cancer development. Using this method, we found that known cancer driver (COSMIC) genes display significantly higher *dN*/*dS* ratios than genes not previously implicated in cancer (i.e., non-COSMIC genes), indicating positive selection for cancer driver gene variants in OC.

Consistent with previous studies [35], we found that *TP53* was the most frequently mutated cancer driver gene in OC (early-stage: 82%; late-stage: 79%; Figure 5). Commonly referred to as “the guardian of the genome”, *TP53* is directly or indirectly involved in a multitude of functions commonly associated with cancer, e.g., cell cycle control, DNA-repair, and programmed cell death (apoptosis) [51]. The second most mutated gene (early-stage 16%; late-stage 6%) shared among our cancer samples was *RNF213*. The function of *RNF213* in cancer remains poorly understood; however, a possible role in the hypoxia-sensitivity of breast cancer cells has recently been reported [52]. Another group of cancer driver gene mutations commonly shared among OC patient samples was the family of *FAT* genes (*FAT3* and *FAT4*). These genes are members of a large family of protocadherins and mutations in both of these genes have been previously implicated in a variety of cancers [53,54], including OC [55,56]. We found that mutations in the *FAT* genes are shared among 26% of early-stage OC patients (*FAT3*: 13%; *FAT4*: 13%). Other than *TP53* and *RNF213*, *FAT3* is the only other gene with mutations commonly present in both early- (13%) and late-stage (7%) OC samples. The remaining most frequent mutated cancer driver genes (Figure 5) found in early-stage samples (*KMT2C* [57], *ALK* [58], *BRCA2* [59], *GRM* [60], *ATM* [61], and *BRCA1* [62]) were not among those found to be the most frequently shared in late-stage samples (*MUC16* [63], *CSMD3* [64], *KNT2C*, *NF1* [65], *LRP1B* [66], *SPEN* [67], and *TRRAP* [68]).

Determining the potential contribution of CNAs to cancer onset and progression is not as straightforward as for nucleotide variation because a multitude of genes are typically associated with these chromosomal regions. We approached this question by utilizing GISTIC2.0 to identify CNAs characterized as “cancer driver regions” based on the number of specific amplification and/or deletions mapping to a chromosomal region and the relative frequency of these events across patient samples. Having identified cancer driver regions in our OC patient samples, we went on to look for significant associations with COSMIC genes mapping to these regions. We found that the proportion of COSMIC genes associated with amplification (AMP) events in cancer driver regions was 4.32%; meanwhile, the proportion in non-driver regions was 3.24%, which is consistent with a model of positive selection for AMP events associated with COSMIC gene sequences in driver regions (Table 2). Interestingly, the proportion of COSMIC genes associated with deletion (DEL) events in driver regions was 2.68%; meanwhile, the proportion in non-driver regions was 3.24%, which is indicative of negative selection for DEL events associated with COSMIC gene sequences in driver regions. When oncogenes (OCGs) were considered alone, the proportion of OCGs associated with AMP events in driver regions was 1.77% while the proportion in non-driver regions was 1.10%; again, this is indicative of positive selection for AMP events associated with OCG sequences in driver regions. The proportion of OCGs associated with DEL events in driver regions was 1.03% while the proportion in non-driver regions was 1.16%; this is indicative of negative selection for DEL events associated with OCG sequences in driver regions.

When TSGs were considered separately, a reversal in the above trends was observed for AMP events. This might have been anticipated because the over-expression of the genes that suppress tumor growth would likely not be favored by developing cancer cells. In accord with this expectation, the proportion of TSGs associated with AMP events in driver regions was 0.71% while the proportion in non-driver regions was 1.21%; this is indicative of negative selection for AMP events associated with TSG sequences in driver regions. While we initially anticipated selection favoring DEL events in regions containing TSGs, no such association was observed. On the contrary, we detected evidence of a slight selection against DEL in the regions associated with TSGs. This may, in part, be due to the fact that CNAs typically encompass many genes and the detrimental effects on cancer cells may override the potential benefits of the DEL of TSGs. In this regard, it may be relevant to note that LOF mutations in many TSGs that are believed to contribute to OC onset/progression (including *TP53*, and *BRCA1/2*) do not map onto cancer driver regions. For these genes, functionally significant mutational changes are occurring on the nucleotide level.

In this era of precision cancer medicine, the focus is often on mutations in specific cancer driver genes associated with individual patient tumors [69]. Such information is certainly critical to the identification of appropriate targeted gene therapies optimized for individual patients; however, the potential clinical value of more generalized patterns of genetic variation associated with individual patient tumors often goes underappreciated. A recent exception is the recognition that relative levels of genetic variation and contributing mutations in DNA-repair genes associated with individual OC and other cancer patient tumors may be a promising predictor of responses to immunotherapy [70].

The clinical significance of levels of genetic variation associated with individual tumors, however, goes well beyond its potential as a biomarker of an immuno-therapeutic response. As was long ago recognized by population geneticists, genetic variation is the sine qua non of adaptive evolution and the same principle applies to the emergence and progression (evolution) of cancers. While it may be obvious that if a cancer driver mutation does not exist in a population of pre-cancerous cells, it cannot be selectively favored in subsequent rounds of replication; not so obvious is the fact that the relative frequency of such mutations in a population of cells can significantly impact the probability that the tumor will successfully develop and, if it does, the frequency at which it will progress. Another relevant consideration is the now well-established fact that individual genes do not operate in a vacuum but are highly dependent upon direct and/or indirect interactions with other genes [71]. Indeed, it is for this reason that well-documented cancer driver mutations are sometimes detected in asymptomatic individuals and patients with the same cancer driver mutation associated with the same cancer type often respond dramatically differently to the same targeted therapy [72].

The fact that we consistently observe a significant increase in levels of genetic variation in patient tumors relative to matched sets of normal cells, is consistent with the hypothesis that genetic variation is a significant contributor to the onset and progression of OC. Our observation that the frequency of functionally significant cancer driver mutations in tumors is greater than the frequency of non-driver mutations is consistent with the view that cancers progress by selecting favorable genetic variants from the pool of variation available to them. This selection is likely not limited to individual cancer driver gene variants but to favorable gene interactions as well.

## 4. Materials and Methods

### 4.1. Data Collection

Genomic data for the study cohort consisted of The Cancer Genome Atlas ovarian cancer samples (TCGA-OV) collected from the Genomic Data Commons (GDC) [18]. Clinical and auxiliary data associated with TCGA-OV samples were retrieved using TCGAbiolinks [73] and TCGAutils [74]. The universal unique identifiers (UUIDs) of the retrieved files can be found in Table S8.

### 4.2. Tumor Mutation Burden (TMB)

To compute tumor mutation burden (TMB), all non-synonymous substitutions and short indels (1–100 bp) found in the cohort-level mutation annotation format (MAF) file were considered. The target regions of the sequencing kit used during the sequencing of each sample in the TCGA cohort were used as an estimate of the coding regions to divide the raw mutation count in order to obtain mutations per megabase (mutations/Mb). For determining the TMB in genes previously implicated in cancer, the coding regions were limited to genes specified in the Cancer Gene Census (CGC) [32] from the Catalogue of Somatic Mutations in Cancer (COSMIC) [75]. To ensure that the number of genes was consistent between the non-COSMIC group and the COSMIC group, bootstrapping was employed to randomly select non-COSMIC genes with the same set size as the number of COSMIC genes. The non-COSMIC gene set was repeatedly sampled using this approach, with the total number of COSMIC genes (723) and the number of oncogenes (OCGs) and tumor suppressor genes (TSGs) (each 243) used as reference points for set size. For each resample during bootstrapping, the TMB for the selected non-COSMIC genes was computed. Subsequently, the median TMB was calculated across all of the resamples to obtain an average TMB value for the non-COSMIC genes for comparison with the COSMIC genes.

### 4.3. Identification of Functionally Significant Mutations

The cohort-level MAF file for TCGA-OV was annotated with Rare Exome Variant Learner (REVEL) [31] scores that were pre-computed for the human genome [76,77] using vcf2maf [78]. A higher REVEL score indicates a greater probability that the annotated variants could be pathogenic. We chose a threshold of 0.38, which demonstrated a balanced performance in minimizing both false positive and false negative results (sensitivity: 0.83; specificity: 0.83).

### 4.4. Analysis of Selection for Genetic Variants

The *dNdScv* R package [34] allowed for a selection analysis that could calculate *dN*/*dS* ratios at both the gene and exome levels. To perform the analysis, the TCGA-OV MAF file was reformatted as an input to compute the ratios for OC samples. For this study, we computed *dN*/*dS* ratio estimates for missense, nonsense, splice site, and overall mutations across the patient exomes. Additionally, mutations were filtered based on the list of COSMIC genes to compute the ratio estimates for COSMIC and non-COSMIC genes to assess patterns of selection in cancer-associated genes.

### 4.5. Identification of Copy Number Alterations

For the gene-level copy number alteration (CNA) data, gain and loss events for the protein-coding genes were defined as amplifications (AMP; 1) and deletions (DEL; −1), respectively. Similar to the procedure for TMB determination, non-COSMIC genes were randomly sampled via bootstrapping in order to compute the median number of observed events for non-COSMIC genes. This allowed for a group-level comparison of the gain and loss events between COSMIC and non-COSMIC genes. Running the Genomic Identification of Significant Targets in Cancer (GISTIC2.0) [41] module on GenePattern [79], with masked copy number segment files, generated output files that include the G-scores for the regions affected by CNAs. The q-values associated with these were used to identify potential driver regions found in OC (q-value < 0.25). Furthermore, GISTIC2.0 provided a list of genes belonging to wide peak regions that undergo more frequent and intense gain/loss events that we referred to as potential driver genes in OC.

### 4.6. Bayesian Analysis

We utilized a Bayesian analytic approach to evaluate how different sample groupings affect TMB and CNA levels. Details of modeling and validation methods have been previously described in published reports [25,26,27]. Briefly, models were implemented using the brms R package [80] to evaluate how different sample stratifications (e.g., early-stage vs. late-stage; COSMIC vs. non-COSMIC) affect TMB and CNA levels. Groups defined by the clinical variables (e.g., stage) and the observed mutations in specified sets of genes (i.e., COSMIC gene type) were considered predictor variables during the modeling process. In the analysis, a reference class was selected to establish a baseline for comparisons with other classes. By default, the package used the first class or group alphabetically as the reference class. This choice provided a consistent framework for evaluating and contrasting the other classes or groups in the analysis. As a response variable, TMB was modeled with either a gamma or a hurdle gamma distribution, depending on whether the presence of zeros needed to be accounted for. For modeling CNAs, a Gaussian distribution was used to model the relationship between the CNAs and the predictor variables. We then computed the fixed-effect estimates for the predictor variables with 95% credible intervals to determine if their effect on the response variable was significant.

## 5. Conclusions

While the results of the analyses regarding the levels of nucleotide variation in ovarian (and other) cancer(s) have been available for over 10 years (e.g., [18,81]), the focus of these earlier studies was, almost exclusively, on the identification of LOF mutations being commonly shared across patient samples putatively identifying drivers of the disease. While our findings are generally consistent with these earlier studies (e.g., *TP53*), we also identified several common gene mutations not previously identified as putative drivers of OC (e.g., *RNY213*, *FAT3/4*). However, the primary focus of our study was not on individual genes but on the significance of levels of genetic variation on the onset and progression of OC. Rather than an analysis of mutations in individual genes, the focus of our study was on the potential clinical significance of overall levels of genetic variation on OC onset and progression. Collectively, our findings indicate that the measurement of relative levels of both nucleotide and chromosomal genetic variation associated with individual OC patient tumors may be a clinically valuable predictor of potential tumor aggressiveness and acquired resistance to chemotherapy. Tumors found to be associated with high levels of genetic variation may help in the clinical identification of high-risk OC patients who could benefit from more frequent monitoring.

Detailed genomic-level analyses of cancer, such as those reported in this paper, have significant benefits and limitations. Among the benefits is the fact that the results often generate novel hypotheses about the processes underlying cancer onset and progression. The limitations are that these hypotheses often require further testing on a clinical level in order to be validated. It is our hope that the findings on OC reported in this paper will stimulate clinical studies leading to improved early diagnosis and treatment of this devastating disease.

## Figures and Tables

**Table 1:** MLEs from *dndscv* for TCGA-OV. The maximum likelihood estimates (MLEs) for the ratios, with their 95% confidence intervals, are shown for each substitution category: missense, nonsense, splice sites, and all substitutions (All). As shown by the columns, these estimates were calculated for COSMIC and non-COSMIC genes (YCOS: COSMIC genes, TSG: tumor-suppressor genes, OCG: oncogenes, NCOS: non-COSMIC genes). Ratios > 1 and <1 indicate positive selection and negative selection, respectively, in cancer.

Stage	Type	YCOS: MLE (95% CI)	TSG: MLE (95% CI)	OCG: MLE (95% CI)	NCOS: MLE (95% CI)
Early	Missense	1.22 (0.95–1.56)	1.18 (0.80–1.75)	1.63 (1.04–2.56)	1.033 (0.97–1.10)
	Nonsense	1.49 (0.93–2.40)	1.99 (1.02–3.87)	1.04 (0.36–3.02)	1.00 (0.87–1.16)
	Splice site	1.00 (0.48–2.08)	1.27 (0.45–3.61)	1.88 (0.65–5.47)	0.50 (0.39–0.65)
	All	1.23 (0.97–1.58)	1.24 (0.84–1.82)	1.61 (1.03–2.53)	1.01 (0.95–1.08)
Late	Missense	1.16 (1.06–1.26)	1.39 (1.21–1.61)	1.02 (0.88–1.19)	1.07 (1.05–1.10)
	Nonsense	1.70 (1.45–1.99)	2.59 (2.07–3.25)	1.20 (0.87–1.64)	1.04 (0.99–1.10)
	Splice site	1.17 (0.93–1.47)	2.12 (1.57–2.86)	0.38 (0.19–0.74)	0.53 (0.49–0.58)
	All	1.19 (1.09–1.30)	1.49 (1.29–1.72)	1.01 (0.87–1.18)	1.05 (1.03–1.08)
All Stages	Missense	1.17 (1.08–1.27)	1.38 (1.20–1.57)	1.08 (0.94–1.25)	1.07 (1.05–1.09)
	Nonsense	1.69 (1.46–1.96)	2.55 (2.06–3.14)	1.19 (0.88–1.61)	1.04 (0.99–1.09)
	Splice site	1.16 (0.93–1.44)	2.03 (1.53–2.70)	0.51 (0.29–0.89)	0.53 (0.49–0.58)
	All	1.20 (1.11–1.31)	1.47 (1.29–1.68)	1.07 (0.93–1.24)	1.05 (1.03–1.08)

**Table 2:** Proportion of COSMIC genes relative to all genes in potential driver regions defined by GISTIC2.0. The number of genes that belong to potential driver regions was counted for both all genes and COSMIC genes. Then, for each alteration type, the proportion of each COSMIC gene category was calculated in relation to all genes in order to examine whether cancer-associated genes were observed more frequently in potential driver regions.

CNA Type	Driver Region	No. of All Genes	No. of All COSMIC	No. of OCGs	No. of TSGs
Amp	No	12,585	408 (3.24%)	139 (1.10%)	152 (1.21%)
	Yes	1133	49 (4.32%)	20 (1.77%)	8 (0.71%)
Del	No	12,552	419 (3.34%)	146 (1.16%)	137 (1.09%)
	Yes	1644	44 (2.68%)	17 (1.03%)	15 (0.91%)

**Table 3:** List of OCGs and TSGs found in GISTIC2 peak regions. Genes listed are accompanied by the type of CNA affecting them (AMP: red; DEL: green) as well as their roles in cancer based on COSMIC (OCG: yellow; TSG; blue). These genes belong to the regions identified by GISTIC2.0 to be relatively higher in frequency and in amplitude for the observed CNA events.

Gene	CNA Type	COSMIC	Gene	CNA Type	COSMIC
*CCNE1*	AMP	OCG	*NF1*	DEL	TSG
*KDM5A*	AMP	OCG	*RAD17*	DEL	TSG
*IKBKB*	AMP	OCG	*LRP1B*	DEL	TSG
*FGFR4*	AMP	OCG	*TSC1*	DEL	TSG
*SDHA*	AMP	TSG	*PTEN*	DEL	TSG
*LARP4B*	AMP	TSG	*MAX*	DEL	TSG
*HRAS*	DEL	OCG	*RAD51B*	DEL	TSG
*HIF1A*	DEL	OCG	*POLE*	DEL	TSG
*MYCN*	DEL	OCG	*FANCA*	DEL	TSG
*CACNA1D*	DEL	OCG	*BAP1*	DEL	TSG
*U2AF1*	DEL	OCG	*PBRM1*	DEL	TSG
*ARID1A*	DEL	TSG	*LATS1*	DEL	TSG
*RB1*	DEL	TSG	*LATS2*	DEL	TSG

## Data Availability

The data presented in this study are available in the Supplementary Materials. Raw data are available at TCGA Research Network: https://www.cancer.gov/tcga (accessed on 14 March 2023).

## Supplementary Materials

The supporting information can be downloaded at: https://www.mdpi.com/article/10.3390/ijms241310823/s1.
