# Supplementary material for: The Clinical Significance of Genetic Variation in Ovarian Cancer
Source: Int J Mol Sci. 2023 Jun 28;24(13):10823. doi: 10.3390/ijms241310823 (PMC10342103; doi:10.3390/ijms241310823)
Supplement: Supplementary file 1 [file ijms-24-10823-s001.zip › Ban Fig S1.pdf]

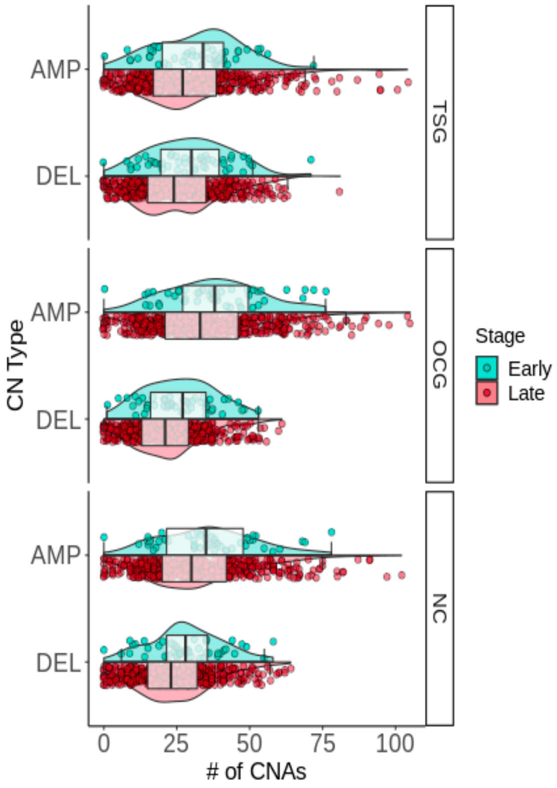

Figure S1: Distribution of CNAs found in COSMIC genes (by subcategories TSG and OCG). The number of observed gene-level CNAs found per sample is shown in violin plots. For comparison, the set size of non-COSMIC genes (NC) was limited to 243 to be consistent with TSG and OCG groups.
